# Multi-dimensional factors and intervention strategies for nasoenteric tube dysfunction: research progress and future perspectives

**DOI:** 10.3389/fmed.2025.1641052

**Published:** 2025-11-28

**Authors:** Xiaoya Zhang, Nana Han, Yan Zhao

**Affiliations:** The Second Affiliated Hospital of Zhejiang Chinese Medical University, Xinhua Hospital of Zhejiang Province, Hangzhou, China

**Keywords:** nasoenteric tube, tube dysfunction, feeding interruption, influencing factor, intervention strategies

## Abstract

Nasoenteric tube (NET) is indispensable for the delivery of enteral nutrition for hospitalized patients. However, their clinical utility is frequently compromised by dysfunction-related complications, including tube malposition, obstruction and displacement. These issues disrupt nutritional therapy, escalate healthcare costs, and exert adverse impact on patient outcomes. Here, we review the multi-factorial etiology of NET dysfunction, encompassing tube-related factors (material properties, structural design and fixation techniques), treatment protocols (nutritional formulation physicochemical properties, drug-nutrient interactions and infusion protocols), nursing practices (flushing practices and errors in the administration of medications) and patient factors (cognitive impairment and non-compliance). To mitigate these issues, we propose intervention strategies, including tube optimization, such as the adoption of polyurethane tubes and guided placement techniques (ultrasound/X-ray) to minimize malposition; protocol standardization (implementing ASPEN guidelines for the administration of medications, including pre-/post-flushing with 30 ml of warm water and avoiding crushed extended-release drugs; multidisciplinary training, such as enhancing the competency of caregivers via education relating to tube maintenance and drug-nutrient compatibility; and patient-tailored strategies, such as using nasal bridle fixation for high-risk patients and sedation protocols for those with cognitive impairment. this review provides a theoretical foundation to optimize the clinical management and efficacy of nutritional therapy. Future research should prioritize the development of risk stratification tools that combine material science, patient factors, and nursing practice to pre-emptively identify high-risk cases and develop integrated scoring systems to evaluate synergistic effects in relation to the cognitive status of patients, tube properties, and nutritional formula viscosity.

## 1 Introduction

The nasoenteric tube (NET), a tube used for short- to medium-term enteral feeding or medication administration when patients at high risk of aspiration or those intolerant to oral or gastric feeding, is inserted through the nasal or oral cavity, traverses the esophagus and pylorus, and terminates in either the duodenum or jejunum, primarily serving for enteral nutritional support (1, 2). Post-pyloric feeding is relatively safe, cost-effective, and capable of maintaining the structural and functional integrity of the intestinal mucosal barrier. This approach can reduce feeding interruptions caused by increased gastric residual volume and also reduce the incidence of reflux pneumonia (3, 4). NET tubes are an essential approach for the establishment of a nutrition pathway for hospitalized patients. Annually, approximately 10% of hospitalized patients worldwide require the use of a NET for enteral nutrition therapy (5).

As a nutritional support pathway for patients, maintaining optimal NET functionality constitutes a fundamental prerequisite for the successful implementation of enteral nutrition. However, in clinical practice, NET may encounter a range of functional complications during clinical placement and utilization, primarily manifesting as tube malposition (e.g., ectopic placement or migration), luminal obstruction, tube fracture (6). A previous study reported that the incidence of tube malposition during NET placement was 14.7%, with 2.1% of these cases progressing to severe complications or mortality (7). Furthermore, the incidence of tube migration or complete dislodgement was 33%, with the risk of luminal obstruction exhibiting a significant and positive correlation with the duration of catheterization (8). The occurrence of NET obstruction is known to range from 25% to 35% and can be caused by the type of NET used, the type of enteral nutrition solution and the administration of drugs by feeding (6, 9). Previous research has indicated that NET obstructions are associated with improper feeding practices and inadequate tube maintenance, particularly when healthcare providers lack sufficient understanding of tube management and care (2, 9–11). These factors can all lead to NET failure and the inability of the NET to function effectively, thus interrupting enteral feeding schedules, potentially leading to inadequate nutritional intake, increased patient discomfort, additional financial burden, an escalation of medical costs, reduced efficiency, and in severe cases, life-threatening adverse outcomes (1, 6–12).

Previous research on NET dysfunction primarily focused on the incidence and risk factors associated with blockage, and the knowledge of nurses with regards to the prevention and management of NET obstructions. Therefore, in this study, we synthesized evidence from clinical studies and guidelines to analyze risk factors and intervention strategies for NET dysfunction including tube-related factors (material and design), treatment protocols (nutrition and drug compatibility), nursing operational standards (flushing and fixation), and patient behaviors (compliance and cognition). Our aim was to enhance the understanding of healthcare professionals with regards to these issues and facilitate the implementation of appropriate preventive measures to ensure successful nutritional therapy outcomes.

## 2 Factors influencing NET dysfunction

### 2.1 NET selection and maintenance

#### 2.1.1 Structural and material factors associated with NET blockage

A NET serves as a nutritional infusion conduit that is inserted via the nasal route, traversing the esophagus and stomach, with its distal tip positioned in either the duodenum or jejunum. The retention length typically exceeds 95 cm; the primary forms include spiral NET, triple-lumen feeding tubes, and capsule jejunostomy tubes (13). These tubes exhibit elongated structural configurations, with diameters ranging from 8 to 12 French (Fr). Contemporary clinical applications primarily utilize nasoenteric tubes fabricated from polyurethane or silicone materials. Medical-grade polyurethane exhibits superior properties when compared to silicone, particularly with regards to the effective infusion of the inner diameter under identical outer diameter conditions, owing to its enhanced elasticity, flexibility, and biocompatibility while maintaining an excellent safety profile. Furthermore, polyurethane tubing is typically engineered with hydrophilic surface coating technology; this enhances surface smoothness and results in significantly higher flow rates during nutritional solution infusion when compared to silicone tubes (14, 15).

#### 2.1.2 Ectopic placement, displacement and the rupture of NET

Malpositioning of a NET represents one of the primary complications associated with enteral tube placement procedures. Multiple factors can influence procedural success, including patient positioning, the level of consciousness, upper gastrointestinal tract anatomy, and operator expertise. These variables may lead to malpositioning of the tube tip, potentially causing patient discomfort, delayed nutritional support, extended hospital stays, and, in severe instances, life-threatening complications (16, 17). While adhesive tape is commonly employed for tube fixation, the adhesive efficacy of such tape can be substantially compromised by facial hair, perspiration, and skin oils, potentially resulting in tube displacement or unintentional removal, thereby disrupting the delivery of enteral nutrition (18). Shi et al. (19) emphasized the importance of considering intestinal peristalsis, particularly retrograde peristalsis, when optimizing external tube fixation while also recommending regular verification of tube position to prevent internal displacement or dislodgement. The duration of NET replacement mainly depends on tube material, empirical evidence (12, 20) also demonstrated a strong and positive correlation between the indwelling duration of a nasoenteric tube and the incidence of lumen obstruction; current guidelines recommend nasoenteric tubes primarily for short-term use, for patients requiring prolonged enteral access, surgically placed gastrostomy tubes represent the alternative. Although tube rupture incidents are uncommon, Fang et al. (21) documented cases associated with spiral NET structural defects and gastric fluid erosion, highlighting the necessity for refined tube maintenance protocols.

### 2.2 Therapeutic factors

#### 2.2.1 Occlusion due to the nature of the nutrient solution and pumping time

Post-pyloric enteral nutrition preparations are typically formulated as whole protein-based or short peptide-based suspensions. Whole protein-based preparations, when exposed to significant pH fluctuations or co-administered with strong electrolytes and alcohols, are susceptible to protein conformational changes, resulting in coagulation and subsequent tube occlusion (22). Furthermore, the introduction of additional drugs into the nutrient solution can destabilize its colloidal system, substantially elevating the risk of tube obstruction. Anziliero et al. (20) identified a significant and positive correlation between the duration of infusion and NET obstruction rates. Prolonged infusion duration leads to increased retention time of the nutrient solution within the tube lumen, causing gradual accumulation and thickening of the adhesion layer on the tube wall. This process exacerbates the aggregation of preparation components, ultimately resulting in solid deposits that significantly increase the risk of tube obstruction.

#### 2.2.2 Tube blockage due to the nature of the drug

The administration of drugs through a NET can readily lead to tube blockage, particularly when solid tablets are inadequately dissolved after grinding or when liquid medications are administered directly with an excessively high concentration and viscosity. Furthermore, an increase in the number of administered medications is known to be positively correlated with a higher incidence of NET blockage (6).

### 2.3 Tube blockage due to healthcare professionals, lack of relevant knowledge and non-standard practices

The physical and chemical properties of nutrient solutions and medications inherently contribute to occlusion risks, proper tube flushing with filtered water is essential to prevent obstruction, and this maintenance protocol requires a multidisciplinary approach involving physicians, pharmacists, nutritionists, and nursing staff. Previous studies (23–26) have indicated that approximately 50% of nursing staff perform tube flushing between enteral drug administrations but lack precise knowledge relating to flushing dosage standards. Furthermore, almost half of healthcare workers incorrectly administer liquid medications without prior dilution, while approximately 21% of nurses add drugs directly to nutritional solutions. Previous surveys (27, 28) revealed that nursing staff generally possess limited theoretical knowledge of enteral medication and demonstrate inadequate attention to nutritional support. These findings reflect system-wide challenges in nasoenteric tube management that extend beyond individual caregiver knowledge and underscore the importance of comprehensive, team-based quality improvement initiatives to optimize quality care for patients using nasoenteric tubes (23, 25–28).

### 2.4 Displacement due to the cognitive behavior of patients

Impaired patient compliance, particularly in cases of cognitive dysfunction, including delirium and dementia, represents a significant risk factor for tube migration (17). Recent evidence (29) support the fact that a range of clinical characteristics, such as a history of stroke, an elevated Glasgow Coma Scale (GCS) score, and advanced age, can significantly increase the risk of nasoenteric tube migration, with a 9% and 2% increase in risk per additional point on the GCS score and per additional year of age. The physical discomfort associated with tube retention can exacerbate psychological distress for both patients and their caregivers, consequently reducing treatment cooperation levels and markedly elevating the complexity of management. Specifically, the incidence of displacement can reach 33% during the initial 72-h period of NET placement (30, 31).

## 3 Intervention strategies to prevent NET malfunction

### 3.1 Tube-related intervention strategies

#### 3.1.1 Selecting the specific type of NET

Boullata et al. (32) previously demonstrated that when considering patient tolerance, prioritizing larger NET lumen diameters for the implementation of enteral nutrition can significantly reduce the incidence of tube obstruction, with the 12Fr model proving more effective than 10Fr or 8Fr models. Han et al. (33) reported that polyurethane tubes with memory spiral tip designs and guidewire devices exhibit significant advantages, as their radio-opaque properties enable complete radiographic visualization, thus facilitating accurate intraoperative positioning and fold identification, thereby providing reliable technical support for the clinical management of infusion obstruction. Niu et al. reported an 89.85% success rate for blind bedside insertion of polyurethane nasogastric tubes, as confirmed by X-ray fluoroscopy, when performed by skilled nurses (34). To address the limitations of traditional NET structures susceptible to obstruction, Kirn et al. (35) developed a vertebral NET with a gradually increasing diameter from the tail to the head end. When compared to tubes with a constant diameter, fluid dynamics research has demonstrated that a NET with a 8–10Fr vertebral design exhibits superior blockage discharge performance, although clinical efficacy requires further validation through evidence-based medical research. Therefore, prior to NET placement, healthcare professionals should select an appropriate tube material and size based on the specific condition of a given patient, along with the clinical setting, to minimize the occurrence of NET dysfunction at its source.

The clinical implementation of advanced tube materials and designs must be carefully weighed against economic considerations, particularly in resource-limited settings. While polyurethane tubes demonstrate superior patency, their higher cost may limit routine adoption in certain healthcare facilities. In such contexts, optimizing the use of more accessible silicone tubes through standardized and rigorously enforced flushing protocols becomes essential. Moreover, blind bedside insertion techniques–when performed by trained and experienced medical staff–remain a critical and cost-effective procedural skill. As evidenced by Niu et al., this approach achieved an 89.85% success rate with polyurethane tubes (34), reinforcing the importance of continuous operator training as a foundational and scalable to reduce malposition risks, even in settings lacking advanced guidance technology.

#### 3.1.2 Flexible NET replacement

The duration of NET replacement should be determined based on the properties of the tube material and the guidelines stipulated in the product specification; the recommended replacement periods are 42 days for polyurethane tubes and 14 days for silicone tubes (33). Research by Yu et al. (36) suggests that extending tube retention time is feasible, provided that safety is ensured. At the same time, Lv et al. (37) demonstrated that a judicious extension of tube use can significantly mitigate mechanical damage from frequent replacement while optimizing cost-effectiveness. For patients requiring long-term enteral nutrition support (typically >4 weeks), percutaneous endoscopic gastrostomy (PEG) should be considered as a safer and more stable alternative to nasoenteric tubes. PEG is easy to install percutaneously using translucency during gastroscopy. The tube needs some care, which is largely standardized and, if necessary, can be easily removed by simple gastroscopy (38, 39). Considering the high technical expertise required for NET placement, and the reluctance of patients and families with regards to frequent replacement, clinicians often adopt a strategy to extend the use of tubes, provided that informed consent is obtained. In particular, when exceeding the recommended replacement cycle, the functional integrity of a tube can be maintained through enhanced nursing care and maintenance protocols, with replacement performed as required to ensure continued safety.

#### 3.1.3 Selection of the NET retention operating technique

Abdominal X-ray is a diagnostic imaging modality that utilizes X-ray technology to visualize the abdominal region. It remains the reference standard for confirming the position of a NET. Due to the complexity and technical challenges associated with NET placement, techniques include blind insertion at the bedside, ultrasound-assisted, endoscopic-guided, magnetic navigation-guided, and X-ray-guided methods. Blind insertion at the bedside, despite its simplicity and immediacy, is associated with certain risks, including tube ectopia or displacement and is characterized by a low success rate. Ultrasound-assisted techniques, as an emerging approach, offer certain advantages, including objectivity, precision, and maneuverability, making these techniques particularly suitable for nursing practice. Wang et al. (40) reported a 95.1% success rate for ultrasound-guided NET placement in critically ill patients at the bedside, a finding consistent with the results of Sun et al. (41) and Bai et al. (42). While endoscopic guidance enhances the success rate, the use of a NET with a 8Fr diameter is often necessitated by the limited size of endoscopic biopsy channels, increasing the risk of blockage. Research indicates that electromagnetic navigation positioning demonstrates 99.5% accuracy when compared with abdominal X-ray results (43). Notably, its accuracy shows no statistically significant difference from endoscopic catheterization (44, 45). However, the clinical application of electromagnetic navigation technology remains limited due to resource constraints. Meanwhile, X-ray guidance–though widely available–faces restrictions in routine use because of radiation exposure risks (46). In clinical practice, blind insertion at the bedside is the preferred initial approach after contraindications are excluded. If unsuccessful, an alternative placement method is selected based on cost-effectiveness and equipment availability to ensure accurate tube positioning. If tube ectopia or displacement is suspected, enteral nutrition should be halted immediately, and the position of the tube tip should be confirmed by abdominal X-ray to facilitate prompt adjustments or reinsertion.

#### 3.1.4 NET fixation techniques

Nasoenteric tube fixation is a critical intervention to prevent displacement, slippage, and unplanned extubation. Conventional fixation methods are often associated with inadequate instability, potentially resulting in tube displacement or unplanned removal. Consequently, clinical nursing researchers worldwide are actively developing more reliable and effective fixation strategies. Notable contributions from Chinese researchers (47–49) have led to a significant enhancement in advanced fixation techniques via the incorporation of flexible tubes and adhesive strips; these advancements have enhanced clinical efficacy, reduced displacement and decannulation rates, and minimized patient discomfort associated with reinsertion procedures. Previous research (50) supported the use of nasal bridle fixation for high-risk patients, demonstrating reduced displacement rates and the improved delivery of enteral nutrition. Standard nursing protocols involve initial nasal fixation using either I-beam or herringbone tape, followed by secondary fixation in the cheek area. Adhesive dressings require routine replacement every 24–48 h and immediate replacement if compromised by moisture or loosening. High-risk patients may benefit from customized secondary fixation protocols to minimize unplanned tube removal. However, the adoption of nasal bridle fixation remains limited in clinical practice. The implementation of evidence-based fixation techniques in clinical practice is hindered by disparities in resource availability. In resource-limited settings where bridles are unavailable, optimizing traditional adhesive fixation through standardized nursing protocols offers a viable workaround. Chinese researchers have pioneered an innovative hybrid approach combining flexible tubing with adhesive strips (47–49), which provides superior stability at minimal additional cost. This modified technique is particularly suitable for large-scale implementation through cascade training models. Ultimately, successful intervention requires more than just improved devices–it demands a systematic approach encompassing three critical components: (1) standardized procedural protocols, (2) enforced dressing change schedules, and (3) nurse empowerment through both training and clinical autonomy. This comprehensive strategy proves most crucial when managing vulnerable patient populations at elevated risk of complications.

### 3.2 Treatment-related interventions

#### 3.2.1 Scientific selection of enteral nutrition formulations

The selection of formulations for enteral nutrition requires comprehensive evaluation based on their compositional characteristics and the specific objectives of nutritional intervention. Current clinical practice predominantly employs suspension-based enteral nutrition preparations (e.g., Enteral Nutritional Suspension, TPF), which maintain stable physicochemical properties, including pH value, viscosity, osmotic pressure, and mineral composition. However, the incorporation of exogenous drugs may disrupt the stability of such solutions, resulting in the formation of a precipitate and an elevated risk of tube occlusion (22). Formulations with high energy density, elevated protein content, or increased fiber concentration exhibit a higher propensity for line occlusion owing to their inherent physicochemical characteristics. When administering dietary fiber-rich or viscous preparations (e.g., Enteral Nutritional Emulsion, TPF-D) after excluding drug contraindications, the connection of a slow drip of 500 ml sterilized water for injection via a “Y” port can effectively dilute the nutrient solution, thereby reducing the probability of occlusion (6, 51). Clinical studies indicate that maintaining an enteral nutrition infusion rate of ≥50 ml/h exerts continuous positive pressure on the nutritional tube, and that sustaining this flow rate within the patient’s tolerance range supports appropriate nutritional fluid flow while minimizing the occurrence of blockage (52). To address the dietary requirements of patients, clinics typically utilize whole protein enteral nutrition preparations administered by a continuous nutrition infusion pump, with dosage determined by the total nutrient solution volume. During the initial enteral nutrition phase, implementing trophic feeding at 20–30 ml/h with corresponding increases in tube flushing frequency is recommended to prevent blockage. As patient tolerance improves, gradual progression to target feeding volumes along with the dynamic optimization of infusion rates can ensure effective enteral nutrition implementation.

#### 3.2.2 Scientific management of oral medication

In the context of nasoenteral drug administration, healthcare professionals are advised to strictly adhere to medication guidelines or consult the prescribing physician while performing a comprehensive evaluation of the physicochemical properties of a given drug, along with crushability and the selected route of administration (32, 53). Before and after the administration of medication, the nutrient tube should be flushed with a minimum of 30 ml of warm water to prevent potential interactions between the drug and enteral nutrition formulations (32). In the context of NET administration, liquid formulations are preferred; however, for concentrated, viscous, or hyperosmolar oral liquids, appropriate dilution should be implemented under the manufacturer’s guidelines. During the sequential administration of multiple medications, the line must be flushed with a minimum of 5 ml of warm water between doses to prevent potential incompatibilities and adverse reactions (22). The American Society for Parenteral and Enteral Nutrition (ASPEN) has established comprehensive guidelines for the administration of tube feeding, encompassing 323 oral medications based on critical parameters including drug solubility, pH, osmolality, clogging potential, required water volume for dissolution, dissolution time, and appropriate flushing volumes (32). When considering non-grindable formulations, including extended-release and controlled-release tablets, Karkossa et al. (54) introduced an innovative delivery technique in which 15 ml of warm water was used to suspend the drug, followed by the injection of 5 ml of air after complete aspiration of the drug solution using a syringe. Following the vertical connection of the syringe to the tube port, rapid 180° rotation was performed, allowing the injection of 7.5 ml of the particle suspension; subsequently, the syringe was returned to the vertical position, and the procedure was repeated to mitigate syringe obstruction. The retained 5 ml air bolus functions as a buffer; in the presence of residual drug particles, the syringe can be tilted to a minus 45° angle to aspirate 15 ml of warm water for subsequent flushing. Following the complete injection of all drug particles, the administration line should be thoroughly flushed with 20–30 ml of warm water. The Simple Suspension Method (SSM) introduced by Kunieda et al. (55) significantly reduced the risk of line obstruction and medication loss when compared to conventional comminution techniques.

For drugs with a high risk of tube blockages, such as linagliptin, mycobacteria, rivaroxaban, and metformin, the current recommendation is to administer the tablets after complete dissolution and filtration through double gauze and to appropriately increase the amount of flushing before and after the administration of the drug to prevent tube blockage (6). While medication crushing remains the most common clinical practice for tube administration, this method requires proper execution to minimize occlusion risks. The use of professional crushing equipment that produces fine, uniform powder is essential, combined with strict adherence to dilution protocols and adequate water flushing between medications (22, 56, 57). Therefore, choosing the appropriate delivery method based on the characteristics of a given drug can effectively avoid the risk of tube occlusion while ensuring therapeutic efficacy. For patients who can tolerate the “dual-tube mode,” a gastric tube can be used for oral drug administration, and a NET can be used for enteral nutrition infusion simultaneously; this strategy can significantly reduce the incidence of tube blockage.

### 3.3 Caregiver-related intervention strategies

#### 3.3.1 Improving multidisciplinary collaboration in NET care

A previous study revealed that systematic interprofessional training programs significantly enhanced team-based medication administration practices; among nursing staff, comprehension of solid dosage form crushing protocols increased from 14% to 63.2%, their knowledge of tube flushing and drug dilution norms improved from 32.6% to 81.6%, and their understanding of tube-feeding medication crushing principles rose from 23.3% to 55.3%. Furthermore, 50% of nursing staff proactively sought the guidance of clinical pharmacists during the administration of medications, demonstrating effective interprofessional collaboration (53, 57). A Jordanian study further corroborated that interprofessional educational interventions significantly enhanced enteral medication safety across the care team (24). While nursing teams play a central role in technical execution of medication and enteral nutrition administration, optimal outcomes require coordinated efforts from physicians, pharmacists, and dietitians. To ensure high-quality care, systematic training programs should therefore be implemented for all healthcare professionals involved in enteral therapy. This collaborative approach enables the development of individualized nutritional therapy plans based on NET type and patient-specific requirements, thereby ensuring the continuous patency of this life-supporting pathway.

#### 3.3.2 Enhanced NET maintenance

Effective tube management during the use of an indwelling NET is essential to maintain optimal function. A comprehensive maintenance procedure must be implemented, including pulsatile flushing with 20–30 ml of luke-warm boiled water at specific time points: before and after nutrient infusion, before and after the administration of medication, and after the interruption of nutritional support (13, 58). From the perspective of hydrodynamics, when liquid flows at low speeds in the tube, its velocity distribution presents the characteristics of maximum flow velocity in the central region and decreasing flow velocity near the wall. Experimental studies by Hayes et al. (59) demonstrated that pulsatile flushing generates eddy currents within the lumen, thereby effectively cleaning the tube walls by preventing the deposition of enteral nutritional fluid or drug particles, thus maintaining lumen patency. Notably, modern, automated, timed and pulsatile nutrient pumps enable the personalized regulation of flushing frequency and dosage, thereby offering enhanced precision in tube maintenance. Subsequent research revealed that a 5% sodium bicarbonate solution exhibited remarkable duct unblocking efficacy, significantly reducing the incidence of nasoenteric duct obstruction (60). Han et al. (33) suggested that systematic maintenance checks of nutrition pumps should be integrated into standard ward management protocols and that the regular maintenance of feeding pumps is essential to ensure accurate delivery rates of enteral nutrition formulas. Furthermore, prompt attention to pump alarms plays a crucial role in the early detection and timely management of tube occlusion. Currently, warm water remains the first-line choice for flushing and sealing feeding tubes in clinical practice. However, there is limited awareness regarding the use of sodium bicarbonate or pancreatic enzymes for flushing in patients with long-term indwelling tubes that are prone to clogging. Furthermore, the potential adverse effects of prolonged use of sodium bicarbonate or pancreatic enzymes require further investigation.

#### 3.3.3 Interventions after NET obstruction

In order to manage NET obstruction, current research primarily recommends the alternating application of pulsatile positive pressure and negative pressure flushing as the standard approach (13). NET obstruction can be managed using a three-way connection device, in which a 10 ml empty syringe and a 10 ml saline syringe are connected to the respective ports of a three-way valve; this enables repeated negative-pressure suction via valve rotation (61). Warm boiled water is recommended as the first-line laxative medium, whereas in refractory cases, sodium bicarbonate solution or pancreatic enzyme preparations may be considered as alternative options. Furthermore, Steinberg et al. (62) reported that the acidic components of Coca-Cola, combined with CO_2_ release and positive pressure effects, could facilitate lumen recanalization. In summary, multiple strategies are available for managing NET obstruction. In cases of severe NET dysfunction, the tube should be promptly removed, followed by a comprehensive evaluation to facilitate safe reintroduction.

### 3.4 Patient-related intervention strategies

The occurrence of adverse events, including tube displacement, ectopic placement, and unplanned extubation, has been demonstrated to be significantly associated with three critical factors: the patient’s level of consciousness (LOC), the degree of muscle strength, and the understanding of family members with regards to tube management (63). For conscious and cooperative patients, healthcare professionals should implement comprehensive patient education programs that emphasize the clinical significance of indwelling tubes and the potential complications associated with premature removal, thereby improving both patient and family knowledge and promoting better treatment compliance. In cases involving patients with dementia, delirium, or other cognitive impairments, clinicians should obtain informed consent from legal guardians and then implement individualized protective measures, including carefully selecting restraint devices based on comprehensive clinical evaluations. In addition to general education and physical restraints, recent evidence supports the use of individualized sedation protocols and behavioral interventions tailored to the patient’s cognitive and clinical profile. For instance, in patients with delirium or dementia, low-dose antipsychotics (e.g., haloperidol or quetiapine) may be considered under psychiatric consultation to reduce agitation and prevent self-extubation, while minimizing sedation-related complications (63). Furthermore, the use of patient-specific communication strategies, such as simplified instructions and visual aids, has been shown to improve cooperation in mildly impaired individuals (16). For high-risk patients with a history of tube dislodgment, the combination of nasal bridle fixation and frequent positional verification via X-ray or ultrasound can significantly reduce displacement rates (18, 50). These approaches should be integrated into a multidisciplinary care plan that includes regular risk assessment and family involvement to ensure both safety and adherence.

## 4 Study limitations

The findings of the present study must be interpreted considering its limitations. It is imperative to critically acknowledge the limitations within the current body of evidence supporting these interventions. Many of the cited clinical studies, particularly those investigating novel tube designs or specific maintenance protocols, are characterized by relatively small sample sizes and are often conducted within single-center settings. The findings obtained through this approach are inherently susceptible to bias, and the absence of multi-center validation significantly limits their generalizability. Furthermore, the predominance of observational or quasi-experimental designs in some areas, such as the evaluation of training programs, introduces a potential for confounding biases, constraining the strength of the recommendations that can be made. Therefore, to robustly validate the efficacy and scalability of these interventions, future research must prioritize large-scale, multi-center, randomized controlled trials (RCTs). Such high-quality studies are essential for definitively establishing the superiority of one intervention over another (e.g., electromagnetic guidance versus ultrasound-guided placement) across diverse patient populations and clinical settings. Beyond comparative effectiveness, future work should also focus on the development and validation of integrated risk stratification tools that synergistically combine material science, patient-specific factors (e.g., cognitive status, diagnosis), and nursing practices. Such tools would enable the pre-emptive identification of high-risk cases and facilitate personalized care plans, ultimately moving the field toward a more predictive and preventive model of NET management.

## 5 Conclusion

As one of the important modes of clinical nutritional therapy, the efficacy of enteral nutritional support via NET is often compromised. In this review, we have summarized the multi-dimensional factors and intervention strategies of NET dysfunction by considering four major themes. First, we considered tube-related factors. Polyurethane tubes reduce obstructions when compared to silicone, while vertebral designs enhance patency and nasal bridle fixation can reduce displacement rates. Second, we considered treatment protocols. For example, implementing the ASPEN guidelines for medication administration, including pre-/post-flushing with 30 ml of warm water and avoiding the use of crushed and extended-release drugs. Third, we considered multi-disciplinary training and the specific enhancement of caregiver competency via education relating to tube maintenance and drug-nutrient compatibility. Finally, we considered patient factors such as the use of nasal bridle fixation for high-risk patients and sedation protocols for cognitively impaired patients.
